# Syntheses of 2-substituted 1-amino-4-bromoanthraquinones (bromaminic acid analogues) – precursors for dyes and drugs

**DOI:** 10.3762/bjoc.11.253

**Published:** 2015-11-26

**Authors:** Enas M Malik, Younis Baqi, Christa E Müller

**Affiliations:** 1 PharmaCenter Bonn, Pharmaceutical Institute, Pharmaceutical Chemistry I, Pharmaceutical Sciences Bonn (PSB), University of Bonn, An der Immenburg 4, D-53121 Bonn, Germany; 2 Department of Chemistry, Faculty of Science, Sultan Qaboos University, PO Box 36, Postal Code 123, Muscat, Oman

**Keywords:** anthraquinone, bromaminic acid, drug synthesis, dyes, intermediates

## Abstract

Anthraquinone (AQ) derivatives play a prominent role in medicine and also in textile industry. Bromaminic acid (1-amino-4-bromoanthraquinone-2-sulfonic acid) is an important precursor for obtaining dyes as well as biologically active compounds through the replacement of the C4-bromo substituent with different (ar)alkylamino residues. Here we report methods for the synthesis of bromaminic acid analogues bearing different substituents at the 2-position of the anthraquinone core. 1-Aminoanthraquinone was converted to its 2-hydroxymethyl-substituted derivative which, under different reaction conditions, yielded the corresponding carbaldehyde, carboxylic acid, and nitrile derivatives. The latter was further reacted to obtain 1-amino-2-tetrazolylanthraquinone. Subsequent bromination using bromine in DMF led to the corresponding bromaminic acid derivatives in excellent isolated yields (>90%) and high purities. Alternatively, 1-amino-4-bromo-2-hydroxymethylanthraquinone could be directly converted to the desired 2-substituted bromaminic acid analogues in high yields (85–100%). We additionally report the preparation of bromaminic acid sodium salt and 1-amino-2,4-dibromoanthraquinone directly from 1-aminoanthraquinone in excellent yields (94–100%) and high purities. The synthesized brominated AQs are valuable precursors for the preparation of AQ drugs and dyes.

## Introduction

Anthraquinones (AQs, anthracene-9,10-diones) represent an important class of organic compounds. They may be produced synthetically, but many derivatives can also be found in nature, e.g., in medicinal plants, as well as in bacteria, fungi and some insects [1–6].

Both, natural and synthetic AQs, are utilized for a wide range of applications, e.g., in textile industry [7–8], paints, foods, cosmetics, pharmaceuticals, and imaging devices [3,9–13], and there is a continuous interest in optimizing this class of compounds as documented in recent literature [14–19]. AQ derivatives can also exert a variety of pharmacological activities including laxative, anti-inflammatory [20–21], antitumor [22–23], antifungal [24], antiviral [25], and blood platelet inhibitory effects [26–28].

**Figure 1:**
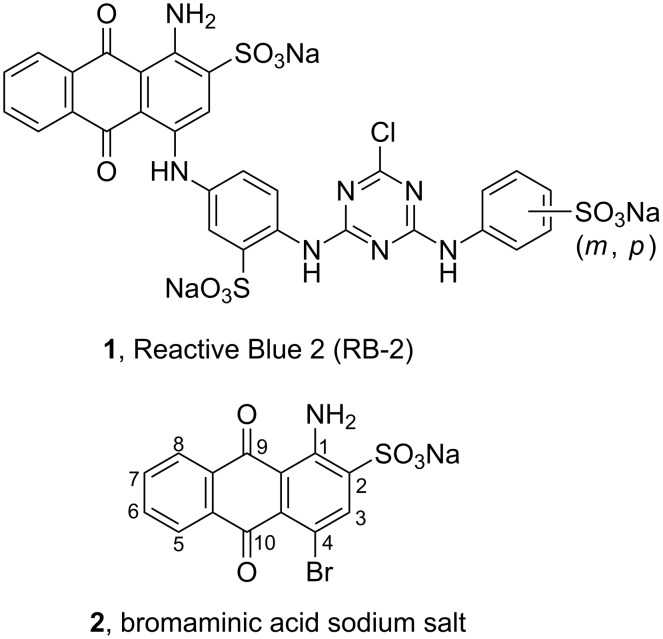
Structures of the anthraquinone derivatives Reactive Blue 2 (RB-2) and bromaminic acid sodium salt.

Reactive Blue 2 (RB-2), a chlorotriazinyl-containing AQ dye, defined as a mixture of two constitutional isomers (**1**, Figure 1), was initially introduced as a colorant in textile industry in the 1950s, and was later found to be useful for the purification of proteins by gel filtration and affinity chromatography techniques [29–32]. In 1979, Kerr and Krantis proposed the compound to interact with ATP-binding sites [33], and it was subsequently used as a pharmacological tool for studying ATP and other nucleotide receptors. RB-2 has played a crucial role in identifying different purine receptor subtypes, since it was found to selectively block only certain members of the nucleotide-activated purine P2 receptor family [34–40]. However, it should be mentioned that ever since the commercially available dye has been used as a tool compound in P2 receptor research, there has been some doubt about its identity and purity, both of which are prerequisites for reliable receptor characterization and subdifferentiation [41].

Our laboratory has a long-standing interest in the development of potent and selective purine receptor antagonists and ectonucleotidase inhibitors as pharmacological tool compounds to study the proteins’ functions and their potential as drug targets. In this context, a library of AQ derivatives, structurally related to RB-2, has been synthesized and evaluated at a variety of purinergic targets; all of which are characterized by a nucleotide binding site [28,42–48]. The nature of the substituent at position 4 of the AQ core was found to be crucial for high affinity of the compounds and also for obtaining selectivity for a specific target [28,43–48]. Investigation of the influence of substituents at position 2 of the AQ derivatives has, however, been very limited due to difficulties in accessing the desired compounds.

**Scheme 1:**
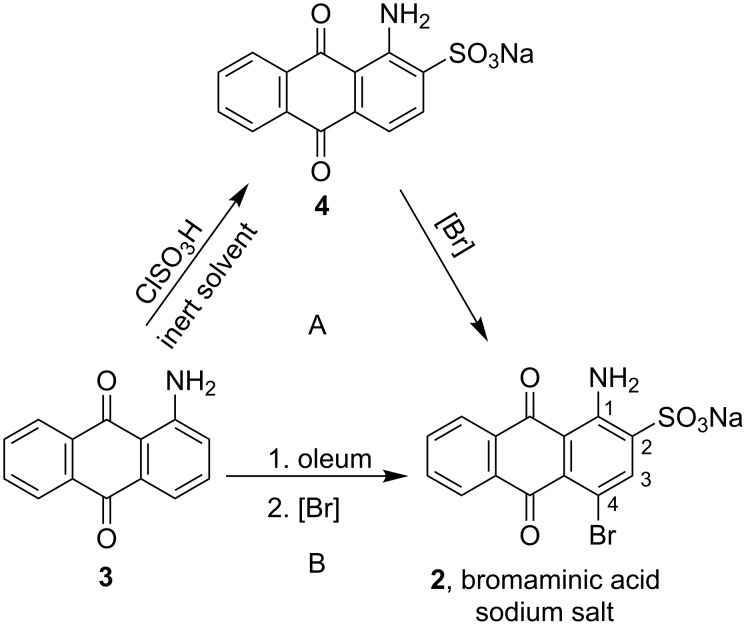
Conventional methods for the synthesis of bromaminic acid sodium salt. (A) solvent method, (B) oleum or one-pot method.

Many of the synthesized AQ derivatives possess a sulfonate group at position 2. They are prepared from sodium 1-amino-4-bromoanthraquinone-2-sulfonate (**2**, Figure 1), i.e., the sodium salt of the commonly known bromaminic acid. This compound represents a key starting material for the synthesis of biologically active AQ derivatives as well as a large number of dyes. In fact, bromaminic acid is one of the most utilized intermediates for the synthesis of AQ derivatives, including acid dyes and reactive dyes, through replacement of the C4-bromine atom by an (ar)alkylamino residue [49]. Procedures for the synthesis of the sodium salt of bromaminic acid are typically described in patents and may be grouped into the so-called “solvent method” and the “oleum method” (Scheme 1). In the solvent method, 1-aminoanthraquinone (**3**) is sulfonated with chlorosulfonic acid in an inert solvent (e.g., nitrobenzene) to form 1-aminoanthraquinone-2-sulfonic acid (**4**), the salt of which is then brominated to give bromaminic acid sodium salt (**2**). Sulfonation of **3** (oleum method) is achieved by the use of oleum followed by bromination in a one-pot reaction. Therefore, the oleum method is also known as “one-pot method” [50].

Introducing different substituents at position 2 of the AQ moiety has been described in a few patents, e.g., [51–55], however, many of them turned out not to be reproducible in our hands. For example, Hagen et al. described harsh reaction conditions to introduce a carboxylate group by oxidizing 2-methylanthraquinone using nitric acid in the presence of nitrobenzene and heating the reaction mixture to 200 °C [54], a procedure which failed in our hands. However, introduction of a bromine or a hydroxymethyl residue in position 2 of 1-aminoanthraquinone was possible following published procedures [56–57] (see below). In the present study we describe convenient optimized methods for the synthesis of various 2-substituted 1-amino-4-bromoanthraquinone derivatives (bromaminic acid analogues) starting from the commercially available 1-aminoanthraquinone (**3**). The synthesized compounds are important intermediates for the production of AQ dyes and for the development of potent and selective ligands for a variety of (potential) therapeutic targets.

## Results and Discussion

**Scheme 2:**
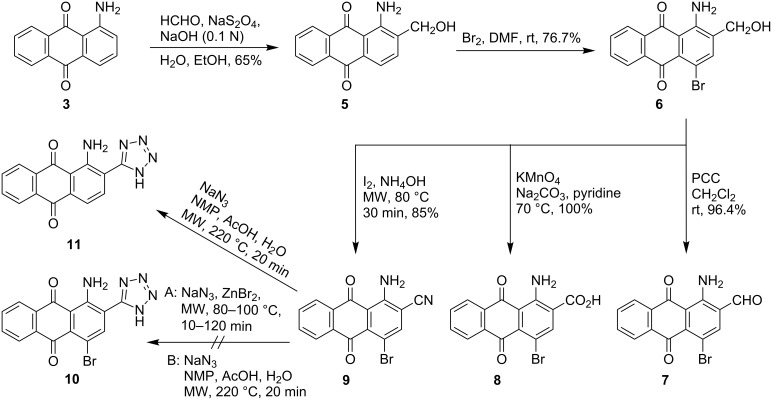
Synthesis of 2-substituted 1-amino-4-bromoanthraquinone derivatives **6–9**.

Following a published procedure, 1-aminoanthraquinone (**3**) was treated with sodium dithionite and formaldehyde under alkaline conditions to yield 1-amino-2-hydroxymethylanthraquinone (**5**) in good isolated yield [56] (Scheme 2). Compound **5** was subjected to bromination using bromine in *N*,*N*-dimethylformamide (DMF). The reaction proceeded smoothly at room temperature affording 1-amino-4-bromo-2-hydroxymethylanthraquinone (**6**) in high yield (Scheme 2 and Table 1).

**Table 1:** Spectral data, melting points, yields, and purities of the synthesized anthraquinone derivatives.

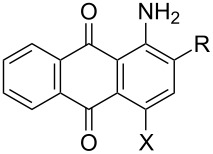								

Compd	R	X	HRMS (g/mol)^a^ *m*/*z*: [M − H]^−^		Absorption λ_max_ (nm)	mp (°C)^b^	Yield (%)^c^	Purity (%)^d^

			Calculated	Found				

**2**	SO_3_Na	Br	379.9228^e^	379.9237^e^	488	270–272	94.0	99.2
**4**	SO_3_Na	H	302.0123^e^	302.0134^e^	482	280	87.0	99.4
**5**	CH_2_OH	H	252.0661	252.0681	486	197–199	65.0	96.0
**6**	CH_2_OH	Br	329.9766	329.9762	480	241–243	76.7	94.0
**7**	CHO	Br	327.9609	327.9602	506	234–236	96.4 (A) 91.3 (B)	99.2 (A) 99.7 (B)
**8**	CO_2_H	Br	343.9558	343.9567	508	315–317	100 (A) 94.3 (B)	96.5 (A) 99.0 (B)
**9**	CN	Br	324.9613	324.9609	486	269–270	85.0 (A) 95.0 (B)	95.0 (A) 97.0 (B)
**10**	tetrazolyl	Br	367.9783	367.9796	506	260–262	92.0	98.0
**11**	tetrazolyl	H	290.0678	290.0693	504	293–296	97.7	99.3
**12**	CN	H	247.0508	247.0523	472	259–261	87.7	95.0
**13**	CHO	H	252.0661^f^	252.0650^f^	494	243–245	82.8	97.8
**14**	CO_2_H	H	266.0453	266.0468	504	292–294	93.6	99.5
**15**	Br	Br	381.8901^f^	381.8899	478	225–227	100	98.1

^a^HRMS was recorded on a micrOTOF-Q mass spectrometer (Bruker) coupled with an HPLC; ^b^The corresponding lit. mp are as follows: compound **5**: 200–201 °C [56], compound **6**: 242 °C [58], compound **7**: 226–228 °C [59], compound **8**: 296–298 °C [60], compound **12**: 260–261 °C [52], compound **13**: 235–238 °C [59], compound **14**: 289 °C [59], and compound **15**: 223 °C [57]; ^c^Isolated yield; ^d^Purity was determined using LC–MS coupled to a UV detector, (A) synthesis from compound **6**, (B) synthesis from the corresponding non-brominated analogue; ^e^*m*/*z*: [M − Na]^−^, ^f^*m*/*z*: [M + H]^+^.

The synthesis of compound **6** had previously been described in the literature by treating bromaminic acid sodium salt (**2**) with sodium dithionite and formaldehyde under alkaline conditions [61]. We initially tried to reproduce the published procedure, however the resulting product contained ca. 10–12% of the debromo derivative **5** which was difficult to separate from product **6**. It should be noted that the commercially available bromaminic acid sodium salt is sold with only ca. 90% purity, and we have previously identified the main contaminant to be the debromo derivative **4** [42], which could be the source for the presence of debromo derivative **5** in product **6**. Using our new approach, the presence of **5** as a contaminant was avoided and we were able to obtain product **6** in high purity starting from commercially available 1-aminoanthraquinone (**3**).

Compound **6** was subsequently used to prepare the corresponding 2-carbaldehyde **7**, the 2-carboxylic acid **8**, and the 2-cyano derivative **9** under the following reaction conditions. Treatment of compound **6** with pyridinium chlorochromate in dichloromethane afforded carbaldehyde **7**. Potassium permanganate was used as the oxidizing agent to produce the corresponding carboxylic acid derivative **8** from alcohol **6**. The reaction was optimized regarding the amount of potassium permanganate to be added and the intervals for addition; it was complete within 50 min affording **8** in quantitative yield. The cyano derivative **9** was prepared by oxidative amination of **6** in the presence of solid iodine in aqueous ammonium hydroxide solution (25%) under microwave (MW) irradiation by optimizing a published procedure [62]. Additional amounts of iodine and ammonium hydroxide were needed to drive the reaction towards completion, while attempts to complete the reaction by increasing the temperature and/or reaction duration were not successful. It was therefore optimal to start with four equivalents of iodine followed by two subsequent additions of two equivalents, each at 10 min intervals, followed by a final 10 min of MW irradiation to complete the reaction. The compounds **7–9** were obtained in excellent isolated yields and purities (see Scheme 2, Table 1).

In the next step we tried to introduce a tetrazolyl moiety at position 2 starting from the cyano derivative **9** using two different previously described approaches [62–63]. According to Shie et al., compound **9** was treated with sodium azide and zinc bromide under MW irradiation [62], however, the desired product 1-amino-4-bromo-2-tetrazolylanthraquinone (**10**, Scheme 2) could not be obtained. Following an alternative procedure, nitrile **9** was reacted with sodium azide in a mixture of *N*-methylpyrrolidone, acetic acid, and water at 220 °C under MW irradiation [63]. However, these conditions led to the loss of the bromine substituent at position 4, yielding compound **11** as the main product (Scheme 2). The debromination might be promoted by the high reaction temperature (220 °C) using acetic acid as the potential hydrogen donor for the reaction [64–65].

**Scheme 3:**
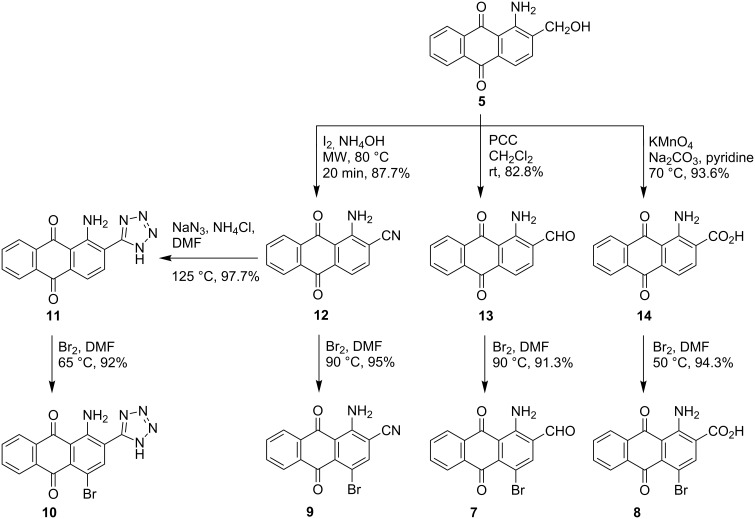
Synthesis of 2-substituted 1-amino-4-bromoanthraquinone derivatives **7–10**.

Therefore, we re-designed our synthetic strategy to start with compounds lacking bromine at position 4 and only introducing the bromine atom in the last step (Scheme 3). Accordingly, compound **5** was converted to 1-amino-2-cyanoanthraquinone (**12**) using the same oxidative amination procedure as described above. A total of six equivalents of iodine was needed to produce the desired product **12** in high yield (Scheme 3, Table 1). The method of Gutmann et al. was subsequently applied to the conversion of nitrile **12** to the corresponding 1-amino-2-tetrazolylanthraquinone (**11**). The conversion was completed within 8 min as determined by TLC, but surprisingly no trace of the desired tetrazole **11** was detected by LC–MS. We presume that the tetrazole was unstable under the applied reaction conditions [66]. Tetrazole derivative **11** was alternatively obtained in excellent yield and purity by refluxing nitrile **12** with sodium azide and ammonium chloride in DMF, followed by treatment with bromine to yield the corresponding brominated tetrazole **10** in excellent yield and high purity (see Scheme 3, Table 1).

The same strategy (bromination in the last step) was subsequently tried for the preparation of the other 2-substituted analogues of bromaminic acid, namely carbaldehyde **7**, carboxylic acid **8**, and nitrile **9**, starting from non-brominated analogues. Thus, starting from compound **5**, and following the oxidation procedures described earlier, we successfully prepared the non-brominated carbaldehyde **13** and carboxylic acid **14**. Subsequently, carbaldehyde **13**, carboxylic acid **14** as well as nitrile **12** were brominated affording the 4-bromoanthraquinone derivatives **7**, **8** and **9** in excellent yields (Scheme 3, Table 1).

Interestingly, using this new strategy, not only were we able to get access to tetrazole **10** in high yield and purity, but also conversion of the hydroxymethyl group of compound **5** to cyano, carbaldehyde, and carboxylate groups yielding compounds **12**, **13**, and **14**, required shorter reaction times, and provided somewhat higher overall yields as compared to the corresponding conversions of the brominated derivative **6**.

**Scheme 4:**
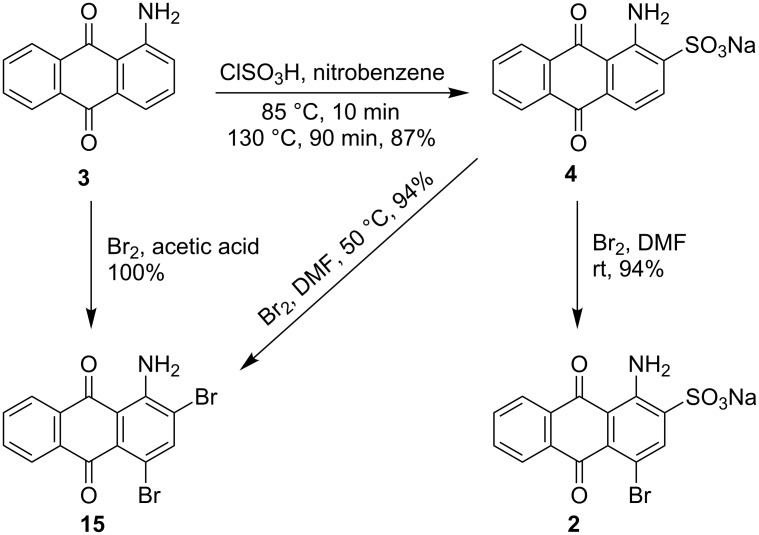
Synthesis of 2-substituted 1-amino-4-bromoanthraquinone derivatives **2** and **15**.

The scope of the procedure was further explored by applying it to the synthesis of bromaminic acid sodium salt (**2**). For this purpose, 1-aminoanthraquinone was reacted with chlorosulfonic acid in nitrobenzene to obtain sodium 1-aminoanthraquinone-2-sulfonate (**4**) [67]. The latter was then treated with bromine in DMF to obtain bromaminic acid as a sodium salt. The sulfonic acid at position 2 of compound **4** is an easily removable group and could be readily replaced by bromine affording 1-amino-2,4-dibromoanthraquinone (**15**) as the sole product. Control of the reaction conditions by conducting the reaction at room temperature with slow addition of a diluted solution of bromine in DMF was very crucial for selective bromination at position 4. Bromaminic acid sodium salt was thus obtained in excellent yield and purity (Scheme 4). 1-Amino-2,4-dibromoanthraquinone (**15**) could alternatively be obtained quantitatively from 1-aminoanthraquinone (**3**) by treatment with bromine under acidic conditions [57] (Scheme 4).

All synthesized compounds were identified by ^1^H NMR, ^13^C NMR and HRMS and additionally characterized by UV absorption and melting points (Supporting Information File 1)

## Conclusion

In conclusion, we successfully introduced seven different substituents (Br, SO_3_Na, CH_2_OH, CHO, CO_2_H, CN, and tetrazole) in position 2 of the anthraquinone moiety starting from commercially available 1-aminoanthraquinone. A general, simple bromination procedure, which provided high yields of the target compounds, was successfully applied to the synthesis of the different target compounds. The described procedure represents a new approach to prepare derivatives possessing a hydroxymethyl or a carbaldehyde group at position 2 of the anthraquinone scaffold. It also provides bromaminic acid sodium salt in high purity, whereas the commercially available product shows a purity of only about 90% and contains ca. 10% of the analogue lacking the bromine atom. Moreover, the described procedures provide access to novel 4-brominated anthraquinone derivatives with a cyano or a tetrazolyl substituent at position 2, as well as to the carboxylic acid analogue of bromaminic acid, the synthesis of which is only described in patents under conditions which proved unsuccessful in our hands. All synthesized compounds were identified and characterized using different spectroscopic methods and found to be highly pure without requiring complicated, time-consuming purification procedures. The synthesized compounds are useful intermediates for the preparation of a variety of anthraquinone derivatives that have potential for different applications, e.g., as colorants in the textile industry, as pharmacological tools for biological investigations, and as potential drugs.

## Supporting Information

Materials and methods, detailed synthetic procedures, and analytical and spectroscopic data of all compounds (**2, 4–15**) are provided.

File 1 Experimental part.
