# Supplementary material for: Syntheses of 2-substituted 1-amino-4-bromoanthraquinones (bromaminic acid analogues) – precursors for dyes and drugs
Source: Beilstein J Org Chem. 2015 Nov 26;11:2326–33. doi: 10.3762/bjoc.11.253 (PMC4685860; doi:10.3762/bjoc.11.253)
Supplement: File 1 — Experimental part. [file Beilstein_J_Org_Chem-11-2326-s001.pdf]

Supporting Information

for

Syntheses of 2-substituted 1-amino-4-bromo-anthraquinones (bromaminic acid analogues) – precursors for dyes and drugs

Enas M. Malik[1], Younis Baqi,*[2] and Christa E. Müller*[1]

Address: [1]PharmaCenter Bonn, Pharmaceutical Institute, Pharmaceutical Chemistry I, Pharmaceutical Sciences Bonn (PSB), University of Bonn, An der Immenburg 4, D-53121 Bonn, Germany and [2]Department of Chemistry, Faculty of Science, Sultan Qaboos University, PO Box 36, Postal Code 123, Muscat, Oman.

Email: Younis Baqi - baqi@squ.edu.om; Christa E. Müller - christa.mueller@uni-bonn.de

* Corresponding author

Experimental Part

Table of Contents

Materials and methods S3

Syntheses and analytical data of compounds (2, 4 – 15) S4

1-Amino-2-hydroxymethyl-9,10-dioxo-9,10-dihydroanthracene (5) S4

1-Amino-4-bromo-2-hydroxymethyl-9,10-dioxo-9,10-dihydroanthracene (6) S5

1-Amino-9,10-dioxo-9,10-dihydroanthracene-2-carbaldehyde (13) S6

1-Amino-4-bromo-9,10-dioxo-9,10-dihydroanthracene-2-carbaldehyde (7) S7

1-Amino-9,10-dioxo-9,10-dihydroanthracene-2-carboxylic acid (14) S8

1-Amino-4-bromo-9,10-dioxo-9,10-dihydroanthracene-2-carboxylic acid (8) S9

1-Amino-9,10-dioxo-9,10-dihydroanthracene-2-carbonitrile (12) S10

1-Amino-4-bromo-9,10-dioxo-9,10-dihydroanthracene-2-carbonitrile (9) S11

1-Amino-2-(1*H*-tetrazol-5-yl)-9,10-dioxo-9,10-dihydroanthracene (11) S13

1-Amino-4-bromo-2-(1*H*-tetrazol-5-yl)-9,10-dioxo-9,10-dihydroanthracene (10) S14

Sodium 1-amino-9,10-dioxo-9,10-dihydroanthracene-2-sulfonate (4) S15

Sodium 1-amino-4-bromo-9,10-dioxo-9,10-dihydroanthracene-2-sulfonate (2) S16

1-Amino-2,4-dibromo-9,10-dioxo-9,10-dihydroanthracene (15) S16

References S17

Materials and methods

All materials were used as purchased (from Acros, Alfa Aesar, Grüssing, Merck, or Sigma-Aldrich, Germany). Thin-layer chromatography was performed using TLC aluminum sheets silica gel 60 F_{254}, or TLC aluminum sheets RP silica gel 18 F_{254} (Merck, Darmstadt, Germany). Colored compounds were visible at daylight; other compounds were visualized under UV light (254 nm). Flash chromatography was performed on a Büchi system using silica gel RP-18 (Merck, Darmstadt, Germany). Microwave reactions were conducted in a CEM Focused^{TM} Microwave Synthesis type Discover apparatus. A freeze dryer (CHRIST ALPHA 1-4 LSC) was used for lyophilization. Melting points were determined using a Büchi Melting Point B-545 apparatus.

The purities of isolated products were determined by ESI–mass spectra obtained on an LCMS instrument (Applied Biosystems API 2000 LCMS/MS, HPLC Agilent 1100) using the following procedure: the compounds were dissolved at a concentration of 0.5 mg/mL in water:methanol (1:1), containing 2 mM NH_4CH_3COO. Then, 10 µL of the sample was injected into an HPLC column (Phenomenex Luna 3µ C18, 50 × 2.00 mm). Elution was performed with a gradient of water:methanol (containing 2 mM NH_4CH_3COO) from 90:10 to 0:100 starting the gradient immediately at a flow rate of 250 µL/min for 15 min followed by washing with 100 % methanol for another 15 min. UV absorption was detected from 200 to 600 nm using a diode array detector. The purity of the compounds proved to be ≥ 94%.

HRMS was recorded on a micrOTOF-Q mass spectrometer (Bruker) with ESI-source coupled with an HPLC Dionex Ultimate 3000 (Thermo Scientific) using an EC50/2 Nucleodur C18 Gravity 3 µm column (Macherey-Nagel). The column temperature was

25 °C. Ca. 1 µL of a 1 mg/mL solution of the sample in acetonitrile was injected and a flow rate of 0.3 mL/min was used. HPLC was started with a solution of acetonitrile in water (10:90), containing 2 mM NH_4CH_3COO. The gradient was started after 1 min reaching 100% acetonitrile within 9 min and then flushed with this concentration for another 5 min. ^{1}H- and ^{13}C-NMR data were collected on a Bruker Avance 500 MHz NMR spectrometer at 500 MHz (^{1}H), and 126 MHz (^{13}C), or on a 600 MHz NMR spectrometer at 600 MHz (^{1}H), and 151 MHz (^{13}C). DMSO-d_6 or chloroform-*d* was used as a solvent. Chemical shifts are reported in parts per million (ppm) relative to the deuterated solvent, i.e. DMSO, δ ^{1}H: 2.49 ppm; ^{13}C: 39.7 ppm; chloroform, *δ* ^{1}H: 7.26 ppm; ^{13}C: 77.36 ppm. Coupling constants *J* values are given in Hertz and spin multiplicities are given as s (singlet), d (doublet), t (triplet), m (multiplet), br (broad).

Syntheses and analytical data of compounds (2, 4 – 15)

1-Amino-2-hydroxymethyl-9,10-dioxo-9,10-dihydroanthracene (5)

Sodium dithionite (2.26 g, 13 mmol) was added under argon to a suspension of finely powdered 1-amino-9,10-dioxo-9,10-dihydroanthracene (**3**) (2.20 g, 10 mmol) in 75 mL of a mixture of ethanol:water:1N NaOH (5:1:1.5, v/v/v). The mixture was then heated at 60 °C until a clear light orange solution was obtained, then it was cooled down to rt followed by the addition of 37 % formaldehyde (8 mL, 100 mmol) under argon with vigorous stirring. The reaction was stopped after 40 s and a stream of air was introduced to the reaction mixture from an air-line. The resulting precipitate was collected by filtration, washed with water and dried under vacuum. Column chromatography of the crude product on silica gel using a gradient of acetone/dichloromethane from 1 % up to 5 %

gave compound **5** (1.65 g, 65.0 %) as a red solid; **m.p**. 197 – 199 °C (lit. m.p. 200 – 201 °C) [1]. **HPLC-UV (254 nm) ESI-MS, purity**: 96.0 %. **LC-MS (*m/z*)**: 252 $[M - H]^-$, 254 $[M + H]^+$. **^{1}H NMR** (500 MHz, DMSO-d_6): δ 4.56 (d, 2H, -CH_2, ^3J 5.45 Hz), 5.47 (t, 1H, -OH, ^3J 5.45 Hz), 7.48 (d, 1H, 4-H, $^3J_{3,4}$ 7.5 Hz), 7.63 (d, 1H, 3-H, $^3J_{3,4}$ 7.5 Hz), 7.84 (td, 1H, 6-H or 7-H, $^3J_{5,6} = {}^3J_{6,7} = {}^3J_{7,8}$ 7.6 Hz, $^4J_{5,7} = {}^4J_{6,8}$ 1.4 Hz), 7.89 (td, 1H, 6-H or 7-H, $^3J_{5,6} = {}^3J_{6,7} = {}^3J_{7,8}$ 7.6 Hz, $^4J_{5,7} = {}^4J_{6,8}$ 1.4 Hz), 8.14 (ddd, 1H, 5-H or 8-H, $^3J_{5,6} = {}^3J_{7,8}$ 7.6 Hz, $^4J_{5,7} = {}^4J_{6,8}$ 1.4 Hz, $^5J_{5,8}$ 0.45 Hz), 8.22 (ddd, 1H, 5-H or 8-H, $^3J_{5,6} = {}^3J_{7,8}$ 7.6 Hz, $^4J_{5,7} = {}^4J_{6,8}$ 1.4 Hz, $^5J_{5,8}$ 0.45 Hz). **^{13}C NMR** (126 MHz, DMSO): δ 60.3 (CH_2), 111.6 (C-9a), 115.5 (C-4), 126.2, 126.4 (C-5, C-8), 132.1 (C-3), 132.4, 132.6, 134.5 (C-8a, C-10a, C-4a), 133.4, 134.3 (C-6, C-7), 134.9, 149.6 (C-1, C-2), 182.7 (C-10), 184.4 (C-9). **HRMS** (ESI-TOF) m/z: $[M - H]^-$ calcd. for $C_{15}H_{10}NO_3$ 252.0661, found 252.0681.

1-Amino-4-bromo-2-hydroxymethyl-9,10-dioxo-9,10-dihydroanthracene (6)

A solution of bromine (0.815 mL, 15.8 mmol) in dry DMF (5 mL) was dropwise added within 60 min to a solution of 1-amino-2-hydroxymethyl-9,10-dioxo-9,10-dihydroanthracene (**5**) (2.0 g, 8 mmol) in dry DMF (40 mL) at rt. After 20 min, an additional amount of bromine (0.103 mL, 2.0 mmol) in DMF (1 mL) was added dropwise resulting in complete reaction (as monitored by TLC). A solution of $NaHSO_3$ (31.0 g) in demineralized water (30 mL) was added and the formed precipitate was filtered off, washed with water, and dried in an oven at 50 °C. The crude product was subjected to column chromatography using a gradient of acetone/dichloromethane from 5 % up to 20 % affording compound **6** (2.01 g, 76.7 %) as a red solid; **m.p**. 241 – 243 °C (lit. m.p. 242 °C) [2]. **HPLC-UV (254 nm) ESI-MS, purity**: 94.0 %. **LC-MS (*m/z*)**: 332 $[M + H]^+$,

334 [M + H + 2]$^+$. **^{1}H NMR** (500 MHz, DMSO-d_6) δ 4.54 (d, 2H, -CH_2, ^3J 5.4 Hz,), 5.57 (t, 1H, -OH, ^3J 5.4 Hz), 7.77 (s, 1H, 3-H), 7.83 (m, 2H, 6-H, 7-H), 8.08 (m, 1H, 5-H or 8-H), 8.14 (m, 1H, 5-H or 8-H). **^{13}C NMR** (126 MHz, DMSO) δ 59.5 (CH_2), 108.2 (C-4), 113.5 (C-9a), 126.1, 126.4 (C-5, C-8), 129.2 (C-2), 133.2 (C-10a), 133.6 (C-8a), 133.7, 134.2 (C-6, C-7), 136.2 (C-4a), 138.2 (C-3), 149.5 (C-2), 182.1 (C-10), 184.0 (C-9). **HRMS** (ESI-TOF) m/z: [M – H]$^-$ calcd. for $C_{15}H_9BrNO_3$ 329.9766, found 329.9762.

1-Amino-9,10-dioxo-9,10-dihydroanthracene-2-carbaldehyde (13)

Pyridinium chlorochromate (1.62 g, 7.5 mmol) was added to a suspension of 1-amino-2-hydroxymethyl-9,10-dioxo-9,10-dihydroanthracene (**5**) (1.27 g, 5 mmol) in dry dichloromethane (62.5 mL). A small amount of celite was added and the mixture was stirred at rt for 15 h. The reaction mixture was then filtered over a pad of celite, washed with dichloromethane, and the filtrate was dried in vacuo. Column chromatography on silica gel (dichloromethane) provided **13** (1.04 g, 82.8%) as a red solid; **m.p**. 243 – 245 °C (lit. m.p. 235 – 238 °C) [3]. **HPLC-UV (254 nm) ESI-MS, purity**: 97.8 %. **LC-MS (*m/z*)**: 250 [M – H]$^-$, 252 [M + H]$^+$. **^{1}H NMR** (500 MHz, Chloroform-*d*): δ 7.66 (d, 1H, 4-H, $^3J_{3,4}$ 7.8 Hz), 7.76 (td, 1H, 6-H or 7-H, $^3J_{5,6}$ = $^3J_{6,7}$ = $^3J_{7,8}$ 7.6 Hz, $^4J_{5,7}$ = $^4J_{6,8}$ 1.35 Hz), 7.82 (td, 1H, 6-H or 7-H, $^3J_{5,6}$ = $^3J_{6,7}$ = $^3J_{7,8}$ 7.6 Hz, $^4J_{5,7}$ = $^4J_{6,8}$ 1.35 Hz), 7.91 (d, 1H, 3-H, $^3J_{3,4}$ 7.8 Hz), 8.25 (dd, 1H, 5-H or 8-H, $^3J_{5,6}$ = $^3J_{7,8}$ 7.6 Hz, $^4J_{5,7}$ = $^4J_{6,8}$ 1.35 Hz), 8.31 (dd, 1H, 5-H or 8-H, $^3J_{5,6}$ = $^3J_{7,8}$ 7.6 Hz, $^4J_{5,7}$ = $^4J_{6,8}$ 1.35 Hz), 9.99 (s, 1H, -CHO). **^{13}C NMR** (500 MHz, Chloroform-*d*): δ 114.2 (C-4), 115.5 (C-9a), 123.4 (C-2), 127.1, 127.2 (C-5, C-8), 132.7 (C-8a or C-10a), 133.7, 134.7 (C-6, C-7), 134.8 (C-8a or C-10a), 138.8

(C-4a), 143.1 (C-3), 152.8 (C-1), 183.3 (C-10), 185.3 (C-9), 193.7 (CHO). **HRMS** (ESI-TOF) m/z: $[M + H]^+$ calcd. for $C_{15}H_{10}NO_3$ 252.0661, found 252.0650.

1-Amino-4-bromo-9,10-dioxo-9,10-dihydroanthracene-2-carbaldehyde (7)

Method A. Pyridinium chlorochromate (0.323 g, 1.5 mmol) was added to a suspension of 1-amino-4-bromo-2-hydroxymethyl-9,10-dioxo-9,10-dihydroanthracene (**6**) (0.332 g, 1.0 mmol) in dry dichloromethane (115 mL). A small amount of celite was added and the resulting mixture was stirred at rt until the reaction was complete (as detected by TLC). Then it was filtered over a pad of celite, and washed with dichloromethane. The filtrate was concentrated to ca. 50 mL in vacuo, washed with water (2× 50 mL), and then brine (20 mL), dried over $MgSO_4$, filtered, and the solvent was removed in vacuo. Column chromatography on silica gel using dichloromethane: cyclohexane (30 – 50 %) provided pure compound **7** (0.318 g, 96.4 %) as a red solid, **HPLC-UV (220 – 550 nm) ESI-MS, purity**: 99.2 %.

Method B. A suspension of 1-amino-9,10-dioxo-9,10-dihydroanthracene-2-carbaldehyde (**13**) (0.754 g, 3 mmol) in dry DMF (20 mL) was heated at 90 °C until a clear, red-colored solution was obtained. A solution of bromine (0.307 mL, 6 mmol) in dry DMF (2 mL) was then slowly added over 15 min and the reaction was complete within 5 min (as monitored by TLC). Upon cooling to rt, a red precipitate was obtained. A solution of $NaHSO_3$ (1.20 g) dissolved in demineralized water (12 mL) was added and the precipitate was filtered off, washed with water and dried in the oven at 50 °C. Compound **7** (0.904 g, 91.3 %) was obtained as red solid. **HPLC-UV (220 – 550 nm) ESI-MS, purity**: 99.7 %;

m.p. 234 – 236 °C (lit. m.p. 226 – 228 °C) [3]. **LC-MS (*m/z*)**: 328 $[M - H]^-$, 330 $[M - H + 2]^-$, 330 $[M + H]^+$, 332 $[M + H + 2]^+$. **^{1}H NMR** (500 MHz, DMSO-d_6) δ 7.88 (m, 2H, 6-H, 7-H), 8.09 (m, 1H, 5-H or 8-H), 8.15 (m, 2H, 5-H or 8-H), 8.36 (s, 1H, 3-H), 9.20 (br, 2H, NH_2), 9.99 (s, 1H, CHO).**^{13}C NMR** (126 MHz, DMSO) δ 104.6 (C-4), 116.7 (C-9a), 123.6 (C-2), 125.7, 126.1 (C-5, C-8), 132.6, 132.9 (C-8a or C-10a), 133.6, 134.0 (C-6, C-7), 135.4 (C-4a), 148.8 (C-3), 150.5 (C-1), 181.8 (C-10), 183.5 (C-9), 193.4 (CHO). **HRMS** (ESI-TOF) m/z: $[M - H]^-$ calcd. for $C_{15}H_7BrNO_3$ 327.9609, found 327.9602.

1-Amino-9,10-dioxo-9,10-dihydroanthracene-2-carboxylic acid (14)

A suspension of 1-amino-2-hydroxymethyl-9,10-dioxo-9,10-dihydroanthracene (**5**) (0.253 g, 1 mmol) in pyridine (2.6 mL) was heated at 70 °C until a clear, red-colored solution was obtained. Then 10 % aq. Na_2CO_3 solution (0.5 mL) was added, and subsequently $KMnO_4$ (0.306 g, 1.94 mmol) was added in portions as follows: 0.126 g of $KMnO_4$ was initially added, followed by 0.08 mg after 10 min and 0.05 g after another 10 min. The reaction was run for another 10 min during which 1.5 mL of pyridine was added. A final amount of $KMnO_4$ (0.05 g) was added, and the reaction was complete after additional 10 min (as monitored by TLC). Subsequently, 0.75 mL of aq. Na_2CO_3 solution (10 %) was added. The formed MnO_2 was filtered off and washed with hot water. Pyridine was removed in vacuo, and the remaining aqueous solution was purified with flash column chromatography on reversed phase (RP-18) silica gel starting with water followed by 5 %, and then 20 % of acetone in water to yield compound **14** (0.25 g, 93.6 %) as a red wine-colored solid; **m.p**. 292 – 294 °C (lit. m.p. 289 °C) [3]. **HPLC-UV (254 nm) ESI-MS, purity**: 99.5 %. **LC-MS (*m/z*)**: 266 $[M - H]^-$, 268 $[M + H]^+$. **^{1}H NMR**

(500 MHz, DMSO-d_6): δ 7.39 (d, 1H, 4-H), 7.85 (t, 1H, 6-H or 7-H), 7.91 (t, 1H, 6-H or 7-H), 8.12 (d, 1H, 5-H or 8-H), 8.2 (d, 1H, 5-H or 8-H), 8.27 (d, 1H, 3-H), 9.16 (br, 2H, NH_2), 13.48 (br, 1H, COOH). **^{13}C NMR** (126 MHz, DMSO): δ 113.1 (C-4), 113.9 (C-9a), 117.2 (C-2), 126.2, 126.5 (C-5, C-8), 132.0 (C-8a or C-10a), 133.6 (C-6 or C-7), 134.2 (C-8a or C-10a), 134.7 (C-6 or C-7), 137.4 (C-4a), 138.5 (C-3), 152.8 (C-1), 168.4 (COOH), 182.6 (C-10), 184.3 (C-9). **HRMS** (ESI-TOF) m/z: $[M - H]^-$ calcd. for $C_{15}H_8NO_4$ 266.0453, found 266.0468.

1-Amino-4-bromo-9,10-dioxo-9,10-dihydroanthracene-2-carboxylic acid (8)

Method A. A suspension of 1-amino-4-bromo-2-hydroxymethyl-9,10-dioxo-9,10-dihydroanthracene (**6**, 0.253 g, 1 mmol) in pyridine (2.6 mL) was heated at 70 °C until a clear, red-colored solution was obtained. Then 10 % aq. Na_2CO_3 solution (0.5 mL) was added followed by the addition of $KMnO_4$ (0.31 g, 1.94 mmol) in portions as follows: 0.126 g of $KMnO_4$ was added initially followed by 0.08 mg after 10 min, and 0.05 g after another 5 min. The reaction was run for additional 20 min during which 1.5 mL of pyridine was added before the final addition of 0.05 mg of $KMnO_4$. The reaction was completed after 15 min (as monitored by TLC). Na_2CO_3 solution (0.75 mL, 10 % in H_2O) was then added, and the formed precipitate of MnO_2 was filtered off and washed with hot water. The filtrate was acidified with HCl (concd.) to ca. pH 1 and the formed precipitate was filtered off, washed with water, and dried in an oven at 50 °C. Compound **8** (0.346 g, 100 %) was obtained as red wine-colored solid. **HPLC-UV (254 nm) ESI-MS, purity**: 96.5 %.

Method B. A suspension of 1-amino-9,10-dioxo-9,10-dihydroanthracene-2-carboxylic acid (**14**) (0.15 g, 0.56 mmol) in dry DMF (3.8 mL) was heated at 50 °C until a clear solution was obtained. Bromine (0.137 mL, 2.26 mmol) was then slowly added, and the reaction was completed after 10 min (as monitored by TLC). A solution of $NaHSO_3$ (0.28 g) dissolved in demineralized water (3 mL) was added to the reaction mixture, which was subsequently extracted with ethyl acetate (3× 75 mL). The combined organic layers were washed with brine (30 mL), and dried over $MgSO_4$. The organic solvent was finally removed under vacuum to produce compound **8** (0.183 g, 94.3 %) as a red wine-colored solid. **HPLC-UV (254 nm) ESI-MS, purity**: 99.0 %;

m.p. 315 – 317 °C (lit. m.p. 296 – 298 °C) [4]. **LC-MS (*m/z*)**: 344 $[M – H]^–$, 346 $[M – H + 2]^–$, 346 $[M + H]^+$, 348 $[M + H + 2]^+$. **^{1}H NMR** (500 MHz, DMSO-d_6): δ 7.86 (m, 2H, 6-H, 7-H), 8.06 (dd, 1H, 5-H or 8-H), 8.13 (dd, 1H, 5-H or 8-H), 8.31 (s, 1H, 3-H). **^{13}C NMR** (126 MHz, DMSO): δ 104.6, 116.0, 118.5 (C-2, C-4, C-9a), 126.0, 126.3 (C-5, C-8), 132.7, 133.2 (C-8a, C-10a), 133.8, 134.3 (C-6, C-7), 134.7 (C-4a), 144.2 (C-3), 152.1 (C-1), 167.1 (COOH), 182.0 (C-10), 183.7 (C-9). **HRMS** (ESI-TOF) m/z: $[M – H]^–$ calcd. for $C_{15}H_7BrNO_4$ 343.9558, found 343.9567.

1-Amino-9,10-dioxo-9,10-dihydroanthracene-2-carbonitrile (12)

Iodine (0.305 g, 1.2 mmol) was added to a mixture of 1-amino-2-hydroxymethyl-9,10-dioxo-9,10-dihydroanthracene (**5**) (0.76 g, 0.3 mmol), aq. ammonium hydroxide solution (25 %, 1.35 mL, 18 mmol), and THF (0.4 mL) in a 10 mL microwave vial. The mixture was stirred for 5 min at rt and then irradiated in the microwave at 90 W, 80 °C for 10 min. Additional amounts of iodine (0.165 g, 0.65 mmol), aq. ammonium hydroxide

solution (25 %, 0.65 mL, 8.7 mmol) and THF (0.4 mL) were added, and the mixture was MW-irradiated for another 10 min under the same conditions. After cooling, 5% aq. $Na_2S_2O_3$ solution (60 mL) was added, and the reaction mixture was subsequently extracted with dichloromethane (3× 75 mL). The combined organic layers were washed with brine (30 mL), dried over $MgSO_4$, filtered, and the solvent was evaporated under reduced pressure. Column chromatography of the crude product on silica gel (dichloromethane) provided **12** (0.652 g, 87.7 %) as an orange solid; **m.p**. 259 – 261 °C (lit. m.p. 260 – 261 °C) [5]. **HPLC-UV (220 – 550 nm) ESI-MS, purity**: 95.0 %. **LC-MS (*m/z*)**: 247 $[M – H]^–$, 249 $[M + H]^+$. **^{1}H NMR** (500 MHz, DMSO-d_6) δ 7.43 (d, 1H, 4-H, $^3J_{3,4}$ 7.9 Hz), 7.89 (t, 1H, 6-H or 7-H, $^3J_{5,6}$ = $^3J_{6,7}$ = $^3J_{7,8}$ 7.5 Hz), 7.93 (t, 1H, 6-H or 7-H, $^3J_{5,6}$ = $^3J_{6,7}$ = $^3J_{7,8}$ 7.5 Hz), 8.04 (d, 1H, 3-H, $^3J_{3,4}$ 7.9 Hz), 8.13 (d, 1H, 5-H or 8-H, $^3J_{5,6}$ = $^3J_{7,8}$ 7.5 Hz), 8.20 (d, 1H, 5-H or 8-H, $^3J_{5,6}$ = $^3J_{7,8}$ 7.5 Hz). ^{13}C NMR (126 MHz, DMSO) δ 102.3(C-2), 113.7 (C-9a), 114.2 (C-4), 116.2 (CN), 126.4, 126.6 (C-5, C-8), 132.1, 133.7 (C-8a, C-10a), 134.1, 134.8 (C-6, C-7), 137.2 (C-4a), 139.9 (C-3), 152.1 (C-1), 182.1 (C-10), 184.4 (C-9. **HRMS** (ESI-TOF) m/z: $[M – H]^–$ calcd. for $C_{15}H_7N_2O_2$ 247.0508, found 247.0523.

1-Amino-4-bromo-9,10-dioxo-9,10-dihydroanthracene-2-carbonitrile (9)

Method A. To a 10 mL microwave vial, 1-amino-4-bromo-2-hydroxymethyl-9,10-dioxo-9,10-dihydroanthracene (**6**) (1.0 g, 0.3 mmol), aq. ammonium hydroxide solution (25 %, 1.35 mL, 18 mmol), iodine (0.305 g, 1.2 mmol) and THF (0.4 mL) were added. The mixture was stirred for 5 min at rt and then irradiated in the microwave oven at 90 W, 80 °C for 10 min. Additional amounts of iodine (0.165 g, 0.65 mmol) and aq. ammonium

hydroxide solution (25 %, 0.65 mL, 8.7 mmol) were added to the reaction mixture, and then irradiated in the microwave for another 10 min under the same conditions. Then, another portion of iodine (0.165 g, 0.65 mmol) and aq. ammonium hydroxide solution (25 %, 0.65 mL, 8.7 mmol) were added and the reaction mixture was irradiated for 10 more min. Then, an aq. solution of $Na_2S_2O_3$ (5%, 60 mL) was added, and the organic materials were extracted with dichloromethane (3× 50 mL). The combined organic layers were washed with brine (3× 30 mL), dried over $MgSO_4$, filtered, and the solvent evaporated under vacuum. Column chromatography of the crude product on silica gel using a gradient of dichloromethane/cyclohexane from 30% up to 60 % provided **9** (0.834 g, 85.0 %) as an orange solid. **HPLC-UV (220 – 600 nm) ESI-MS, purity**: 95.0 %.

Method B. A suspension of 1-amino-9,10-dioxo-9,10-dihydroanthracene-2-carbonitrile (**12**, 0.62 g, 2.5 mmol) in dry DMF (20 mL) was heated at 90 °C until a clear, red-colored solution was obtained. A solution of bromine (0.258 mL, 5 mmol) in dry DMF (2 mL) was dropwise added within 30 min. The reaction mixture was stirred at 90 °C for 30 min, before an additional amount of bromine (0.10 mL, 2.0 mmol) in DMF (1 mL) was added dropwise. The progress of the reaction was monitored by TLC, and the reaction was complete within 5 min. Upon cooling down to rt, the product precipitated as an orange-colored solid, then a solution of $NaHSO_3$ (1 g) dissolved in demineralized water (10 mL) was added, and the precipitate was filtered off, washed with water and dried in an oven at 50 °C. Compound **9** (0.777 g, 95.0 %) was obtained as an orange solid. **HPLC-UV (220 – 600 nm) ESI-MS, purity**: 97.0 %;

m.p. 269 – 270 °C. **LC-MS (*m/z*)**: 325 $[M – H]^–$, 327 $[M – H + 2]^–$, 327 $[M + H]^+$, 329 $[M + H + 2]^+$. **^{1}H NMR** (500 MHz, DMSO-d_6) δ 7.89 (m, 2H, 6-H, 7-H), 8.07 (m, 1H, 5-

H or 8-H), 8.13 (m, 1H, 5-H or 8-H), 8.31 (s, 1H, 3-H). **^{13}C NMR** (126 MHz, DMSO) δ 103.6 (C-2), 105.4 (C-4), 114.7 (C-9a), 115.6 (CN), 126.1, 126.4 (C-5, C-8), 132.8 (2C, C-8a, C-10a), 134.2, 134.4 (C-6, C-7), 134.8 (C-4a), 145.5 (C-3), 151.4 (C-1), 181.7 (C-10), 183.8 (C-9). **HRMS** (ESI-TOF) m/z: $[M - H]^-$ calcd. for $C_{15}H_6BrN_2O_2$ 324.9613, found 324.9609.

1-Amino-2-(1*H*-tetrazol-5-yl)-9,10-dioxo-9,10-dihydroanthracene (11)

A 25 mL round bottom flask was fitted with 1-amino-9,10-dioxo-9,10-dihydroanthracene-2-carbonitrile (**12**) (0.248 g, 1.0 mmol), NaN_3 (0.325 g, 5.0 mmol) and NH_4Cl (0.267 g, 5.0 mmol), followed by dry DMF (5 mL) under argon. The mixture was subsequently heated to 125 °C. The color of the solution changed from orange to red wine-color and the reaction was completed after 30 min (as monitored by TLC). It was left to cool down to rt, and it was then slowly added to 30 mL of a saturated $NaHCO_3$ solution. The resulting solution was extracted with ethyl acetate (3× 100 mL). Since part of the product was observed in the organic layer (as detected by TLC), the combined ethyl acetate layers were washed 4–5 times with water (100 mL each) until no product remained in the organic layer (as shown by TLC). $NaNO_2$ (0.27 g) was added to the combined aqueous layers which were then acidified cautiously with conc. HCl to pH 1. The formed precipitate was collected by filtration, washed with water, and dried in an oven at 50 °C to yield product **11** (0.285 g, 97.7 %) as a red solid; **m.p**. 293 – 296 °C. **HPLC-UV (220 – 600 nm) ESI-MS, purity**: 99.3 %. **LC-MS (*m/z*)**: 290 $[M - H]^-$, 292 $[M + H]^+$. **^{1}H NMR** (500 MHz, DMSO-d_6): δ 7.55 (d, 1H, 4-H, $^3J_{3,4}$ 7.7 Hz), 7.85 (t, 1H, 6-H or 7-H), 7.91 (t, 1H, 6-H or 7-H), 8.16 (d, 1H, 5-H or 8-H), 8.27 (d, 1H, 5-H or 8-H),

8.51 (d, 1H, 3-H, $^{3}J_{3,4}$ 7.7 Hz), 9.64 and 9.84 (2s, 1H each, NH_2).**^{13}C NMR** (126 MHz, DMSO): δ 112.4 (C-9a), 115.3 (C-4), 123.1 (C-2), 126.1, 126.5 (C-5, C-8), 132.2 (C-8a or C-10a), 132.3 (C-3), 132.6 (C-8a or C-10a), 133.3, 134.3 (C-6, C-7), 134.7 (C-4a), 149.3 (C-1), 159.3 (C-5´), 182.6 (C-10), 184.1 (C-9). **HRMS** (ESI-TOF) m/z: $[M – H]^{–}$ calcd. for $C_{15}H_8N_5O_2$ 290.0678, found 290.0693.

1-Amino-4-bromo-2-(1*H*-tetrazol-5-yl)-9,10-dioxo-9,10-dihydroanthracene (10)

A suspension of 1-amino-2-(1*H*-tetrazol-5-yl)-9,10-dioxo-9,10-dihydroanthracene (**11**) (0.905 g, 1.75 mmol) in dry DMF (10 mL) was heated at 70 °C until a clear solution was obtained. The temperature was reduced to 65 °C. Bromine (0.18 mL, 3.5 mmol) in 2 mL DMF was dropwise added over 10 – 15 min. A TLC performed immediately after the addition indicated completion of the reaction. The mixture was cooled to rt, and a solution of $NaHSO_3$ (0.7 g) dissolved in demineralized water (8 mL) was added. The product precipitated as a red-colored solid which was filtered off, washed with water, and dried in an oven at 50 °C yielding 0.595 g (92.0 %) of **10** as a red solid; **m.p**. 260 – 262 °C. **HPLC-UV (220 – 600 nm) ESI-MS, purity**: 98.0 %. **LC-MS (*m/z*)**: 368 $[M – H]^{–}$, 370 $[M – H + 2]^{–}$, 370 $[M + H]^{+}$, 372 $[M + H + 2]^{+}$. **^{1}H NMR** (500 MHz, DMSO-d_6): δ 7.87 (m, 2H, 6-H, 7-H), 8.09 (dd, 1H, 5-H or 8-H), 8.17 (dd, 1H, 5-H or 8-H), 8.51 (s, 1H, 3-H). **^{13}C NMR** (126 MHz, DMSO): δ 106.0, 115.5, 115.5 (C-2, C-4, C-9a), 126.1, 126.7 (C-5, C-8), 132.4, 132.8, 133.3 (C-4a, C-8a, C-10a), 133.8, 134.3 (C-6, C-7), 140.4 (C-3), 148.8 (C-1), 154.0 (C-5´), 181.9 (C-10), 183.8 (C-9). **HRMS** (ESI-TOF) m/z: $[M]^{–}$ calcd. for $C_{15}H_7BrN_5O_2$ 367.9783, found 367.9796.

Sodium 1-amino-9,10-dioxo-9,10-dihydroanthracene-2-sulfonate (4)

1-Amino-9,10-dioxo-9,10-dihydroanthracene (**3**) (2.23 g, 10 mmol) was dissolved in nitrobenzene (250 mL) and heated at 120 °C for 15 min to remove traces of water. The solution was then cooled to 85 °C, followed by dropwise addition of chlorosulfonic acid (0.864 mL, 13 mmol) within 10 min. The reaction mixture was subsequently heated at 130 °C for 1.5 h. When the reaction was complete (as monitored by TLC), it was cooled down to rt before the addition of 0.64 g of Na_2CO_3. Ca. 300 mL water was added to the reaction mixture, which was subsequently extracted with diethyl ether (3× 500 mL) to remove nitrobenzene. The water layer was then concentrated in vacuo and subsequently purified by flash column chromatography using reversed phase (RP-18) silica gel, starting with water followed by a gradient of 5% then 20 % of acetone / water to yield compound **4** (2.80 g, 87.0 %) as orange solid; **m.p**. 280 °C. **HPLC-UV (220 – 600 nm) ESI-MS, purity**: 99.4 %. **LC-MS (*m/z*)**: 302 $[M – Na]^–$, 304 $[M – Na + 2H]^+$, 321 $[M – Na + NH_4 + H]^+$. **^{1}H NMR** (600 MHz, DMSO-d_6) δ 7.41 (d, 1H, 4-H, $^3J_{3,4}$ 7.7 Hz), 7.84 (td, 1H, 6-H or 7-H, $^4J_{6,8} = {}^4J_{5,7}$ 1.2 Hz, $^3J_{5,6} = {}^3J_{6,7} = {}^3J_{7,8}$ 7.7 Hz), 7.91 (td, 1H, 6-H or 7-H, $^4J_{6,8} = {}^4J_{5,7}$ 1.2 Hz, $^3J_{5,6} = {}^3J_{6,7} = {}^3J_{7,8}$ 7.7 Hz), 7.95 (d, 1H, 3-H $^3J_{3,4}$ 7.7 Hz), 8.14 (dd, 1H, 5-H or 8-H, $^4J_{6,8} = {}^4J_{5,7}$ 1.2 Hz, $^3J_{5,6} = {}^3J_{7,8}$ 7.7 Hz), 8.23 (dd, 1H, 5-H or 8-H, $^4J_{6,8} = {}^4J_{5,7}$ 1.2 Hz, $^3J_{5,6} = {}^3J_{7,8}$ 7.7 Hz). **^{13}C NMR** (151 MHz, DMSO) δ 112.6 (C-9a), 114.2 (C-4), 126.2, 126.5 (C-5, C-8), 132.4 (C-2), 132.8, 133.5 (C-6, C-7), 134.1 (C-8a or C-10a), 134.5 (C-3), 134.6 (C-8a or C-10a), 138.2 (C-4a), 148.5 (C-1), 182.7 (C-10), 184.0 (C-9). **HRMS** (ESI-TOF) *m*/*z*: $[M – Na]^–$ calcd. for $C_{14}H_8NO_5S$ 302.0123, found 302.0134.

Sodium 1-amino-4-bromo-9,10-dioxo-9,10-dihydroanthracene-2-sulfonate (2)

A suspension of sodium 1-amino-9,10-dioxo-9,10-dihydroanthracene-2-sulfonate (**4**) (0.325 g, 1.0 mmol) in dry DMF (8 mL) was heated to 50 °C until a clear red solution was obtained, then it was cooled down to rt. A solution of bromine (0.103 mL, 2.0 mmol) in DMF (1 mL) was dropwise added within 30 min. After 1 h, monitored by TLC, an additional amount of bromine (0.026 mL, 0.5 mmol) was added to complete the reaction. Then a solution of $NaHSO_3$ (4.0 g) in demineralized water (2 – 3 mL) was added and the reaction mixture was subsequently extracted with dichloromethane (4× 50 mL). The water layer was concentrated in vacuo and purified by flash column chromatography using reversed phase (RP-18) silica gel starting with water followed by 5 % and then 20 % of acetone / water to yield compound **2** (0.380 g, 94.0 %) as an orange solid; **m.p**. 270 – 272 °C. **HPLC-UV (220 – 600 nm) ESI-MS, purity**: 99.2 %. **LC-MS (*m/z*)**: 380 $[M – Na]^-$, 382 $[M – Na + 2]^-$, 382 $[M – Na + 2H]^+$, 384 $[M – Na + 2H + 2]^+$. ^{1}**H NMR** (500 MHz, DMSO-d_6) δ, 7.86 (m, 2H, 6-H, 7-H), 8.04 (s, 1H, 3-H), 8.09 (m, 1H, 5-H or 8-H), 8.16 (m, 1H, 5-H or 8-H).^{13}**C NMR** (126 MHz, DMSO) δ 106.0 (C-4), 114.6 (C-9a), 126.0, 126.3 (C-5, C-8), 131.2 (C-2), 132.9, 133.5 (C-8a or C-10a), 133.6, 134.2 (C-6, C-7), 138.5 (C-4a), 138.8 (C-3), 148.2 (C-1), 182.0 (C-10), 183.5 (C-9). **HRMS** (ESI-TOF) m/z: $[M – Na]^-$ calcd. for $C_{14}H_7BrNO_5S$ 379.9228, found 379.9237.

1-Amino-2,4-dibromo-9,10-dioxo-9,10-dihydroanthracene (15)

1-Amino-9,10-dioxo-9,10-dihydroanthracene (**3**) (1.10 g, 5 mmol) was dissolved in 20 mL glacial acetic acid, the solution was heated to 40 °C, and bromine (11 mL, 10 mmol) was then dropwise added. The resulting mixture was stirred at this temperature for 16 h

followed by 8 h at 100 °C. The reaction was stopped by pouring it into 50 mL of a $NaHSO_3$ solution (40 % in H_2O) to form a dark orange precipitate of product **15**. The precipitate was filtered off, washed with water and dried overnight in an oven at 75 °C to yield 1.90 g (100 %) of compound **15** as a dark orange solid; **m.p**. 225 – 227 °C (lit. m.p. 223 °C) [6]. **HPLC-UV (254 nm) ESI-MS, purity**: 98.1 %. **LC-MS (*m*/*z*)**: 380 $[M – H]^–$. **^{1}H NMR** (500 MHz, DMSO-d_6) δ 7.87 (m, 2H, 6-H, 7-H), 8.09 (m, 1H, 5-H or 8-H), 8.16 (m, 1H, 5-H or 8-H), 8.20 (s, 1H, 3H). **^{13}C NMR** (126 MHz, DMSO) δ 107.2 (C-4), 114.6 (C-9a), 117.2 (C-2), 126.3, 126.5 (C-5, C-8), 130.5 (C-4a), 133.1 (2C, C-8a, C-10a), 134.1, 134.4 (C-6, C-7), 143.6 (C-3), 148.4 (C-1), 181.9 (C-10), 183.8 (C-9). **HRMS** (ESI-TOF) *m*/*z*: $[M + H]^+$ calcd. for $C_{14}H_8Br_2NO_2$ 381.8901, found 381.8899.

References

1. Bredereck, K; Banzhaf, L; Koch, E. *Chem. Ber*. **1972**, *105*, 1062–1071.
2. Metwally, S.A.; Youssef, M.S.; Younes, M.I. *J. Chem. Techol. Biotechnol.* **1980**, *30*, 513–5 19.
3. Bredereck, K; Kimmich, H.; Sigmund, G. *Liebigs Ann. Chem*. **1977**, 184–188.
4. Scholl, R. *Monatsh. Chem*. **1913**, *34*, 1011–1026.
5. Junge, H.; Sturm, H-J.; Gnadbeck-Seeger, H-J. Verfahren zur Herstellung von aromatischen o-Aminonitrilen. DE Patent 2049161, Apr. 13, 1972.
6. Ghaieni, H.; Sharifi, M.; Fattollahy, M. *Dyes Pigment.* **2006**, *71*, 73–76.
